# Differential Biphasic Transcriptional Host Response Associated with Coevolution of Hemagglutinin Quasispecies of Influenza A Virus

**DOI:** 10.3389/fmicb.2016.01167

**Published:** 2016-08-03

**Authors:** Himanshu Manchanda, Nora Seidel, Markus F. Blaess, Ralf A. Claus, Joerg Linde, Hortense Slevogt, Andreas Sauerbrei, Reinhard Guthke, Michaela Schmidtke

**Affiliations:** ^1^Research Group Systems Biology and Bioinformatics, Leibniz Institute for Natural Product Research and Infection Biology - Hans Knoell InstituteJena, Germany; ^2^Department of Virology and Antiviral Therapy, Jena University HospitalJena, Germany; ^3^Integrated Research and Treatment Center - Center for Sepsis Control and Care, Jena University HospitalJena, Germany; ^4^Department of Anaesthesiology and Intensive Care Medicine, Research Unit Experimental Anesthesiology, Jena University HospitalJena, Germany; ^5^Centre of Innovation Competence (ZIK) Septomics, Jena University HospitalJena, Germany

**Keywords:** computational biology, gene expression, transcriptome, viral pathogenicity, immunopathogenesis, pneumonia, mouse models, pandemic H1N1

## Abstract

Severe influenza associated with strong symptoms and lung inflammation can be caused by intra-host evolution of quasispecies with aspartic acid or glycine in hemagglutinin position 222 (HA-222D/G; H1 numbering). To gain insights into the dynamics of host response to this coevolution and to identify key mechanisms contributing to copathogenesis, the lung transcriptional response of BALB/c mice infected with an A(H1N1)pdm09 isolate consisting HA-222D/G quasispecies was analyzed from days 1 to 12 post infection (p.i). At day 2 p.i. 968 differentially expressed genes (DEGs) were detected. The DEG number declined to 359 at day 4 and reached 1001 at day 7 p.i. prior to recovery. Interestingly, a biphasic expression profile was shown for the majority of these genes. Cytokine assays confirmed these results on protein level exemplarily for two key inflammatory cytokines, interferon gamma and interleukin 6. Using a reverse engineering strategy, a regulatory network was inferred to hypothetically explain the biphasic pattern for selected DEGs. Known regulatory interactions were extracted by Pathway Studio 9.0 and integrated during network inference. The hypothetic gene regulatory network revealed a positive feedback loop of Ifng, Stat1, and Tlr3 gene signaling that was triggered by the HA-G222 variant and correlated with a clinical symptom score indicating disease severity.

## Introduction

Influenza A viruses (IAV) cause pandemics with high morbidity and mortality (Webster and Govorkova, 2014). The last influenza pandemic in 2009 caused by IAV of subtype H1N1 (A(H1N1)pdm09) was rather mild with a cumulative incidence of 24% (Van Kerkhove et al., 2013). However, severe influenza cases causing an estimated number of 201,200 respiratory deaths and additional 83,300 cardiovascular deaths in otherwise healthy adults and pregnant woman showed the potential pathogenicity of A(H1N1)pdm09 (Dawood et al., 2012). In search of the cause of severe influenza cases, quasispecies (group of viruses related by one or more mutations) of A(H1N1)pdm09 differing in amino acid in position 222 (H1 numbering) in the viral HA were detected (Chen et al., 2010; Kilander et al., 2010; Liu et al., 2010; Vazquez-Perez et al., 2013; Wedde et al., 2013). Results of a systematic review of 18 published studies, from all continents, revealed that “D222G was associated with a significant increase in severe disease (pooled RD: 11%, 95% CI: 3.0–18.0%, *p* = 0.004) and the risk of fatality (RD: 23%, 95% CI: 14.0–31.0%, *p* = 0.0001). No association was observed between the mutations HAD222N, D222E, PB2-E627K, and NS1-T123V and severe/fatal disease” (Goka et al., 2014). No virus quasispecies bearing virulence-conferring mutations in the HA, PB2, and NS1 predominated but, hemagglutinin (HA) variants of A(H1N1)pdm09 including also HA residue 222 persisted across several seasons (Bedford et al., 2015; Caglioti et al., 2016).

HA-D222G was also detected after adaptation of A(H1N1)pdm09 isolates in mice (Ilyushina et al., 2010; Seyer et al., 2012; Song et al., 2013). Experimental evidence for the association of coevolution of HA-222D/G quasispecies of the A(H1N1)pdm09 isolate Jena/5258/09 (Jena/5258) and severe influenza with biphasic pathology was provided by us recently (Seidel et al., 2014). The increasing amounts of the HA-G222 variant in the lung (~8% at day 1 p.i. and up to ~27% at day 5 and 6 p.i.) of BALB/c mice after infection with the once mouse lung-passaged Jena/5258 (mpJena/5258) (i) coincided with increasing lung virus titers, (ii) preceded severe lung inflammation, and (iii) were associated with a severe symptom peak on day 6 after infection (Seidel et al., 2014). Further adaptation mutations in Jena/5258 as well as mpJena/5258 were excluded by sequencing the eight viral genome segments (Seidel et al., 2014). Infection of BALB/c mice with plaque-purified mpJena/5258 HA-G222 induced strong symptoms with a maximum at day 6 p.i. (Seidel et al., 2014). Therefore, we hypothesize that the observed symptoms are at least partly provoked by the host response to the observed intra-host viral evolution.

In human as well as mice, the highly dynamic and complex influenza virus-host interactions can be accompanied by a strong inflammatory immune response (Zou et al., 2013; Jin et al., 2014; Shoemaker et al., 2015) and excessive lung damage (Kilander et al., 2010; Shieh et al., 2010; Zheng et al., 2010; Vazquez-Perez et al., 2013; Wedde et al., 2013; Seidel et al., 2014). Comprehensive genome wide expression data involving both innate as well as adaptive immune response help to understand molecular mechanisms of host response during severe influenza. Global gene expression changes have been studied using microarray techniques in lungs of mice after infections with different IAV subtypes and variants causing mild or severe disease (Ding et al., 2008; Pommerenke et al., 2012; Askovich et al., 2013; Zou et al., 2013; Shoemaker et al., 2015). Most studies have focused on innate immune responses of the host. To the best of our knowledge, only one study integrated both innate and adaptive immune responses, and describes different phases of disease as temporal changes in gene expression profile in lungs of mice (Pommerenke et al., 2012). Recently, we used mathematical modeling to explain the different course of influenza (mild to severe with mono and biphasic disease dynamics) caused by three A(H1N1)pdm09 isolates based on clinical score data in BALB/c mice (Manchanda et al., 2014). Our modeling results suggest (i) maximum primary pathogenicity, (ii) viral infection rate, and (iii) rate of activation of the immune system as most important parameters that are associated with the different pattern of virus-specific influenza kinetics. Additional experimental studies with one of these A(H1N1)pdm09 isolates, the mpJena/5258, demonstrated that fast intra-host evolution of HA-222D/G quasispecies in BALB/c mice with increasing amounts of the HA-G222 variant in lung and trachea tissue coincide with lung histopathology and disease severity on days 6 and 7 p.i. (Seidel et al., 2014). These results prompted us to study dynamics of host response to the observed quasispecies coevolution to get further insights into the molecular mechanism underlying the observed course of disease during coevolution of HA-222D/G quasispecies.

A comprehensive analysis of whole genome expression changes of lung samples obtained from BALB/c mice infected with mpJena/5258 that consists HA-222D/G quasispecies (Seidel et al., 2014) was performed. The time-dependent production of two key inflammatory cytokines, interferon (IFN-γ), and interleukin 6 (IL-6), which have been shown to be increased during severe influenza infection (Pommerenke et al., 2012; Goraya et al., 2015; Shoemaker et al., 2015) was confirmed by ELISA. The use of lung and serum samples, collected recently (Seidel et al., 2014), enables us to link the pre-existing knowledge on quasispecies coevolution and disease dynamics with genome expression changes in the lung. Finally, a reverse engineering approach was applied to infer a gene regulatory network to predict hypothetically the driving forces for the biphasic pattern—pars pro toto—for a set of 20 DEGs.

## Materials and methods

### Animal experiment

All trial procedures and animal care activities were conducted in accordance with the German Animal Protection Law. Experiment was approved by the Thueringer Landesamt fuer Verbraucherschutz (Reference number: 02-032/12). The animal experiment and the dynamics of disease (severity, lung histopathology, and virological data) were described recently (Seidel et al., 2014). Briefly, 7- to 8-week-old female BALB/c mice (16–18 g; Charles River, Bad Sulzfeld, Germany) were infected intranasally with 10^6^ TCID_50_/20 μl of mpJena/5258 under isoflurane anesthesia. Mock-infected mice were used as control. Five or ten mpJena/5258-infected mice were sacrificed on day 1–7, 9, and 12 p.i. Severe lung inflammation was detected (Seidel et al., 2014). During this experiment Seidel et al. also stabilized one of the right lung lobes in RNAlater solution (Ambion by Life technologies, Darmstadt, Germany) for subsequent RNA analysis. RNA analysis of these frozen lung samples (−80°C) was performed with each 4 of these lung samples of infected mice per time point (d1, 2, 3, 4, 5, 6, 7, 9, and 12 p.i.) by using microarrays. Furthermore, sera of the same mice were used for cytokine detection. RNA (*n* = 4) as well as serum samples (*n* = 3) of uninfected mice were analyzed for control.

### RNA extraction

RNA isolation was performed with the RNeasy Mini Kit (Qiagen, Hilden, Germany) according to the manufacturer's instructions. Afterwards, the amount of RNA was measured with a NanoDrop ND-1000 spectrophotometer (Peqlab, Erlangen, Germany).

### Microarray analysis

Prior to gene expression experiments, total RNA integrity was confirmed using the Experion™ automated gel electrophoresis system (BioRad, Munich, Germany). cRNA sample preparation for hybridisation on the Illumina gene expression platform was performed using the TargetAmp™ Nano-g™ Biotin-aRNA Kit for the Illumina System (Epicentre/Biozym, Hess. Oldendorf, Germany) starting with 250 ng of total RNA. Samples were hybridized according to manufacturer's instructions on Mouse Ref-8 v2.0 Bead Chips (Illumina, San Diego, USA). Each chip comprises probes of 25,700 coding and non-coding RNA transcripts. Read outs of hybridisation signal intensities were performed on an iScan Bead Array scanner (Illumina, San Diego, USA), data pre-processing including spot detection, gene mapping and averaging of replicates was performed with iScan Control Software and GenomeStudio software (Illumina, San Diego). The data are accessible through Gene Expression Omnibus series [GSE67241].

### Microarray data analysis and gene regulatory network inference

**Figure 1:**
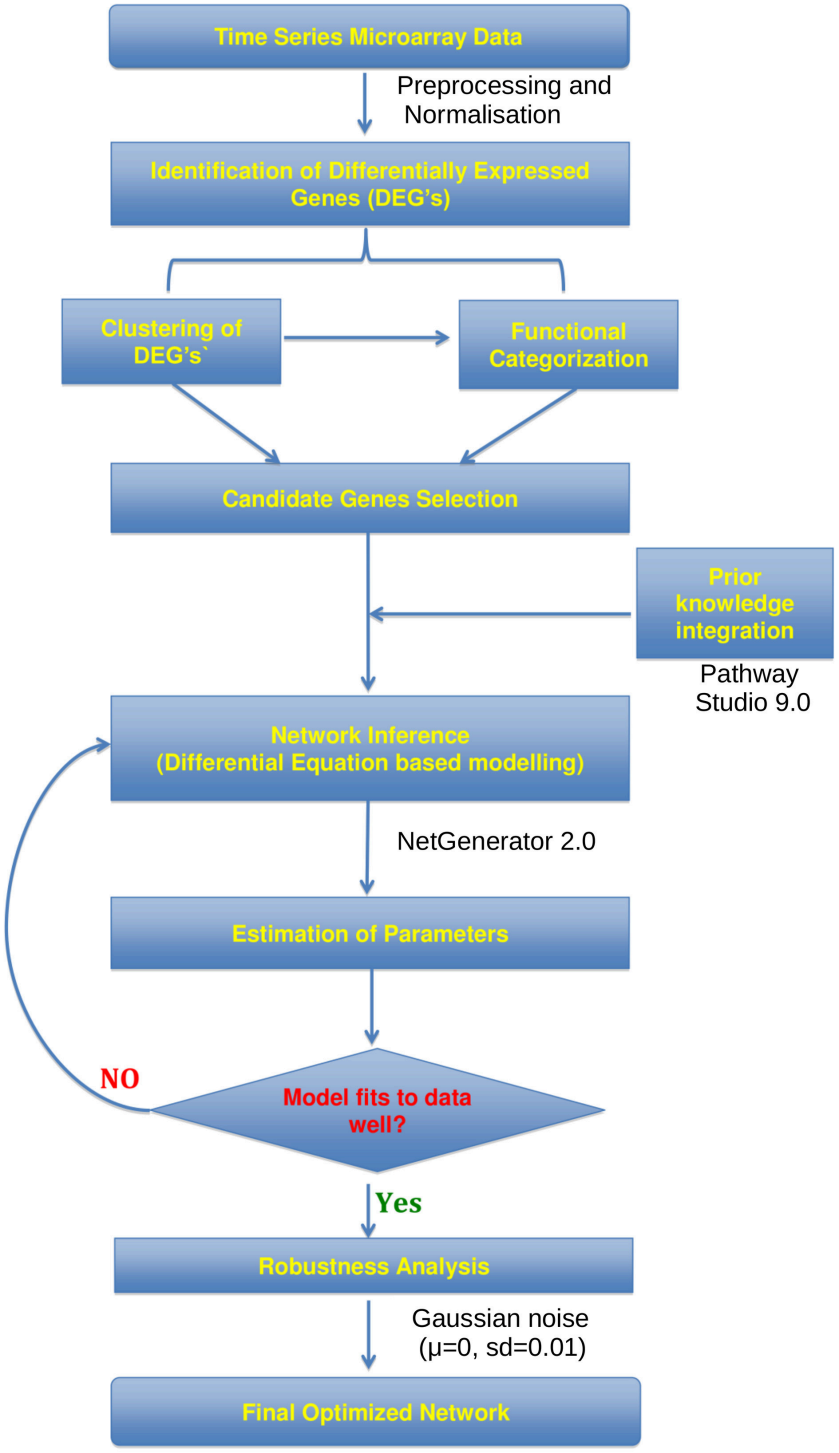
**Workflow**. Flowchart showing the analysis of microarray time series data utilized for the construction of gene regulatory networks using the NetGenerator tool.

The workflow for gene expression data analysis constructing gene-regulatory network from time series microarray data is visualized in Figure 1. Raw microarray data was analyzed using the Lumi (Du et al., 2008) and Limma (Smyth, 2005) packages of the statistical language R (Team RDC, 2009). Between-array normalization was performed using Variance Stabilization and Normalization (vsn) (Lin et al., 2008) with lumiN so that the distribution of intensities should become independent of the mean. DEGs were identified from lung samples of virus-infected compared with mock-infected mice sacrificed on days 7 and 12 (*t* = 0) p.i., using Empirical Bayes statistics with a false discovery rate of 0.01. In order to group genes with similar expression profile we performed fuzzy c-means (Bezdek et al., 1984) clustering to DEG's expression matrix. The optimal number of clusters was estimated as previously described (Guthke et al., 2005) based on 42 cluster validity indices.

Network inference was performed using NetGenerator package implemented in the statistical programming language R. The algorithm is described in detail by Guthke et al. (2005) and Linde et al. (2015). Briefly, it is based on a set of linear differential equations and models the temporal change of expression intensity of the gene *x*_*i*_(*t*) at time *t* as the weighted sum of the expression intensity of all the other genes and two external stimuli *u*_1_(*t*) and *u*_2_(*t*):

$$\frac{dx_{i}}{dt}=\sum_{j\;=\;1}^{n}w_{i,j}x_{j}\left(t\right)+\sum_{k\;=\;1}^{2}b_{i,k}u_{k}\left(t\right)f\;or\;i=1..n$$

The NetGenerator tool calculates the parameters *w*_*i, j*_ and *b*_*i, k*_. The parameter *w*_*i, j*_ represents an influence of gene *j* on the expression of gene *i*, while the parameter *b*_*i, k*_ represents the impact of external stimulus given by the function *u*_k_(*t*). The external stimuli (input perturbation) *u*_1_(*t*) and *u*_2_(*t*) are modeled from the viral titre data for two quasispecies A(H1N1)pdm09 strains taken from Seidel et al. (2014), as shown in Supplementary Figure 1. A non-zero weight *w*_*i, j*_ defines an interaction (edge) of the inferred network where a positive weight is interpreted as activation and a negative one represents the repression or inhibition.

As the number of possible networks structure increases exponentially with the number of genes, a small number of DEGs was selected to be included in the network reconstruction algorithm. In order to identify key genes, significantly overrepresented categories (i.e., *p* < 0.05) were identified using the tool DAVID (Huang da et al., 2009a,b). DAVID is a web based functional annotation tool which provides a comprehensive set of functional enrichment for a given gene list. It uses the Fischer exact statistical test to measure the significance of gene enrichment in annotation terms. Several studies showed that the inclusion of prior knowledge extracted from various literature and other database sources improves the reliability of the network inference approach (Hecker et al., 2009; Tierney et al., 2012; Weber et al., 2013; Linde et al., 2015). The prior knowledge used for network reconstruction extracted from Pathway Studio 9.0 (Nikitin et al., 2003) includes relations only of type “direct interaction” as well as “expression.” Confidence scores were assigned to prior knowledge interactions each used for the network inference based on the source of the data. High score 0.5 was given for ≥70 of supporting literature references, low score 0.1 for between 1 and 10 of supporting literature references and median score 0.25 for numbers of references between 10 and 70. The prior knowledge was softly (i.e., flexibly) integrated during the network inference. Since different data sources might be contradictory, it is advantageous to softly integrate them during the modeling process. The robustness of inferred network edges was investigated by adding Gaussian noise of mean 0 and standard deviation (sd) of 0.01 or 0.1 and repeated network inference procedure for 500 times. The robustness of the inferred edges was quantified by the recovery rate (frequency of repeated inference divided by 500).

### Linear regression model for association between genotype and phenotype

A multiple linear regression model was developed with aim of finding an association between the observed phenotype data, which was quantified by the symptom score *S*, from Manchanda et al. (2014), and the gene expression data:

$$S=\sum_{i\in G}\beta_{i}x_{i}$$

The function “lm” was used to perform multiple linear regression in R. For the gene set *G* = {Ifng, Stat1, Tlr3, Eif2ak2}, the multiple linear regression in the Wilkinson and Roger's notation looks like:

fit = lm(S~ Ifng + Stat1 + Tlr3 + Eif2ak2 − 1). Here the response data *S* was collected from Manchanda et al. (2014). The explanatory data *x*_*i*_ were collected from the current study.

### Cytokine detection in serum

Mouse IFN-γ ELISA (BioLegend, SanDiego, USA) and mouse IL-6 ELISA (eBioscience, SanDiego, USA) were applied according to the manufactures instructions to determine the amounts of these cytokines in serum.

### Statistical analysis

Pairwise significant differences between cytokine values (detected at different days p.i.) were calculated in IBM SPSS statistics 22 with ANOVA (Kruskal-Wallis).

## Results

### Global transcriptome analysis reveals biphasic host response against severe A(H1N1)pdm09 infection

**Figure 2:**
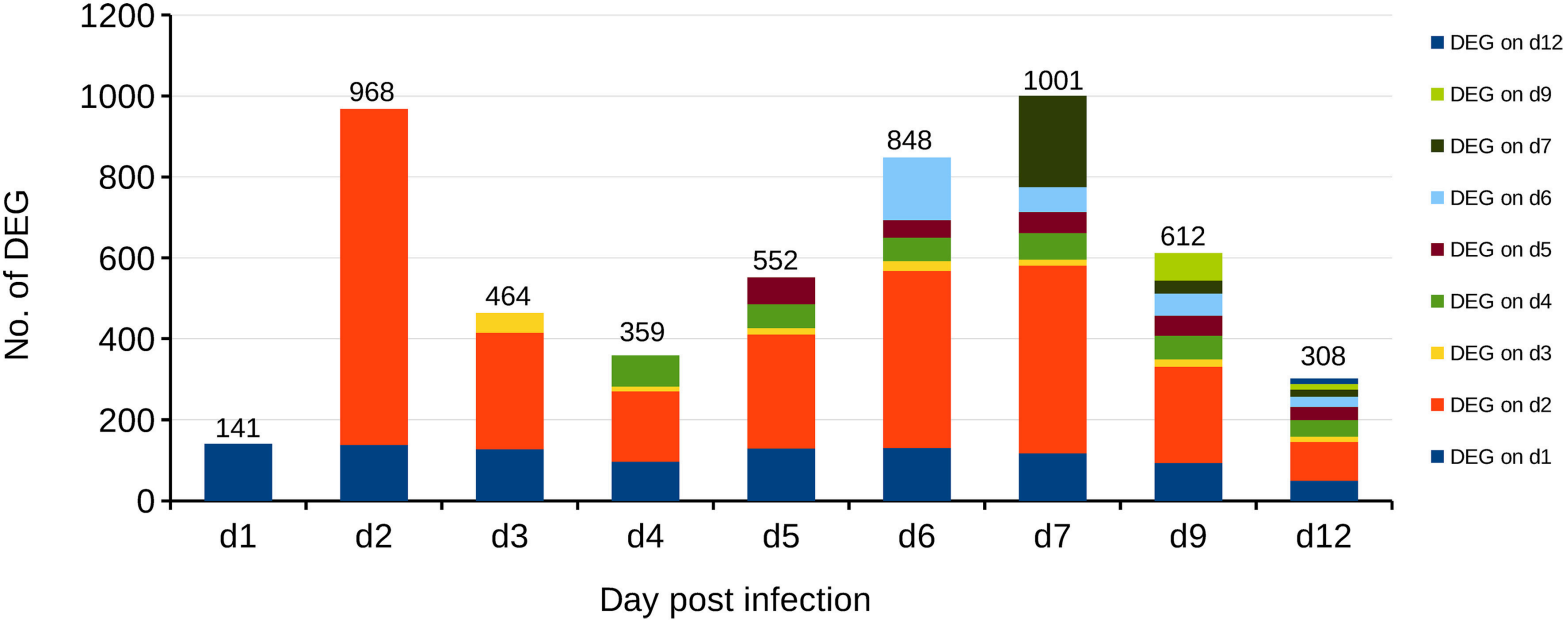
**Differentially Expressed Genes (DEGs) in the lungs of BALB/c mice infected with the once mouse lung-passaged influenza virus A/Jena/5258/09**. The biphasic pattern of gene expression consists of the two peaks at days 2 and 7 after infection. Overall 1628 DEGs were identified between days 1 and 12 after infection. Bars indicate the number of DEGs on each day p.i. Different colors indicate the number of DEGs that were newly detected at the indicated days after infection compared to control. For example, dark blue represents the DEGs, which are present at day 1 p.i. and continue to be present through the infection process with different frequency while the orange color at day 2 p.i., represents DEGs that appeared at day 2 p.i. and were not deferentially expressed before.

Recently, coevolution of HA-222D/G variants was detected in lungs and trachea of BALB/c mice infected with the clinical isolate Jena/5258 as well as it's once mouse lung-passaged variant mpJena/5258 and linked with severe biphasic influenza (Seidel et al., 2014). In the present study, we isolated the RNA of lung samples collected from mpJena/5258-infected BALB/c mice (Seidel et al., 2014) and performed gene expression profiling to get an insight into the dynamics of host response to this severe biphasic viral infection. Thus, the course of disease (body weight changes, clinical score, viral replication, and quasispecies coevolution) of studied mice was well known (Seidel et al., 2014). RNA of lung samples of mock-infected mice was included as control. The gene expression data was normalized and in total 1628 DEGs were identified during the whole infection process (Figure 2; Supplementary Table 1).

**Figure 3:**
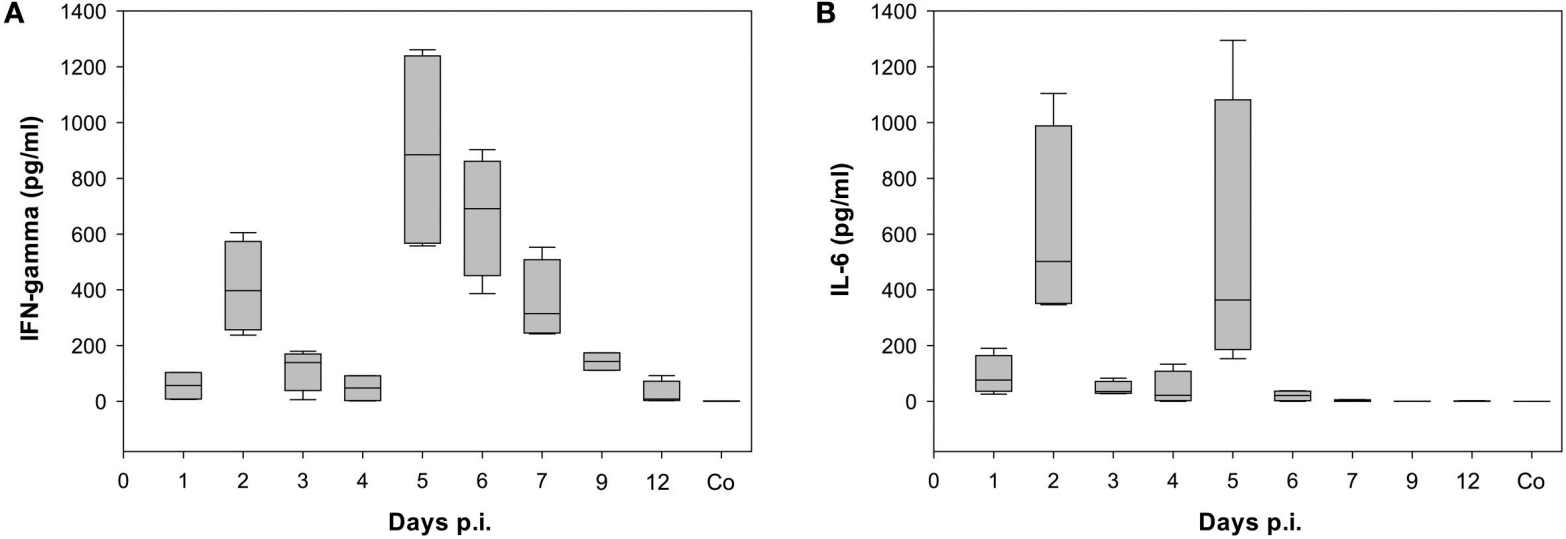
**Time dependence of (A) Interferon gamma (IFN-gamma) and (B) Interleukin 6 (IL-6) levels detected in serum of BALB/c mice infected with the once mouse lung-passaged influenza virus A/Jena/5258/09**. Both cytokines were detected in serum samples of each 4 mice per time point by ELISA. Box blots show the distribution of cytokine values. The values are summarized in Supplementary Table 3 together with the results of statistical analysis.

Already at day 2 p.i., 968 genes were differentially expressed (up- or down-regulated) with respect to control data indicating a strong activation (Figure 2). Genes that are strongly expressed during this early phase of infection include cellular factors involved in the detection of virus-associated molecular pattern (Supplementary Table 2). They belong to Gene Ontology (GO):0009615, “response to virus” (Ashburner et al., 2000; Gene Ontology, 2015). The number of up- and/or down-regulated DEGs in response to infection decreased markedly to about 400 DEGs compared to the control at day 4 p.i. A second peak of an increased transcriptional response was observed with 1001 DEGs at day 7 p.i. Finally, a decrease of the number of regulated DEGs to 308 at day 12 p.i. was observed. Many of the detected DEGs follow the biphasic pattern in relation with the biphasic clinical symptoms score [e.g., changes of fur and behavior after virus infection as described recently (Manchanda et al., 2014; Seidel et al., 2014)]. In accordance the biphasic course of the inflammatory immune response was reflected by the levels of the proinflammatory cytokines IFN-γ and IL-6 both detected by ELISA in serum of infected animals (Figure 3; Supplementary Table 3).

**Figure 4:**
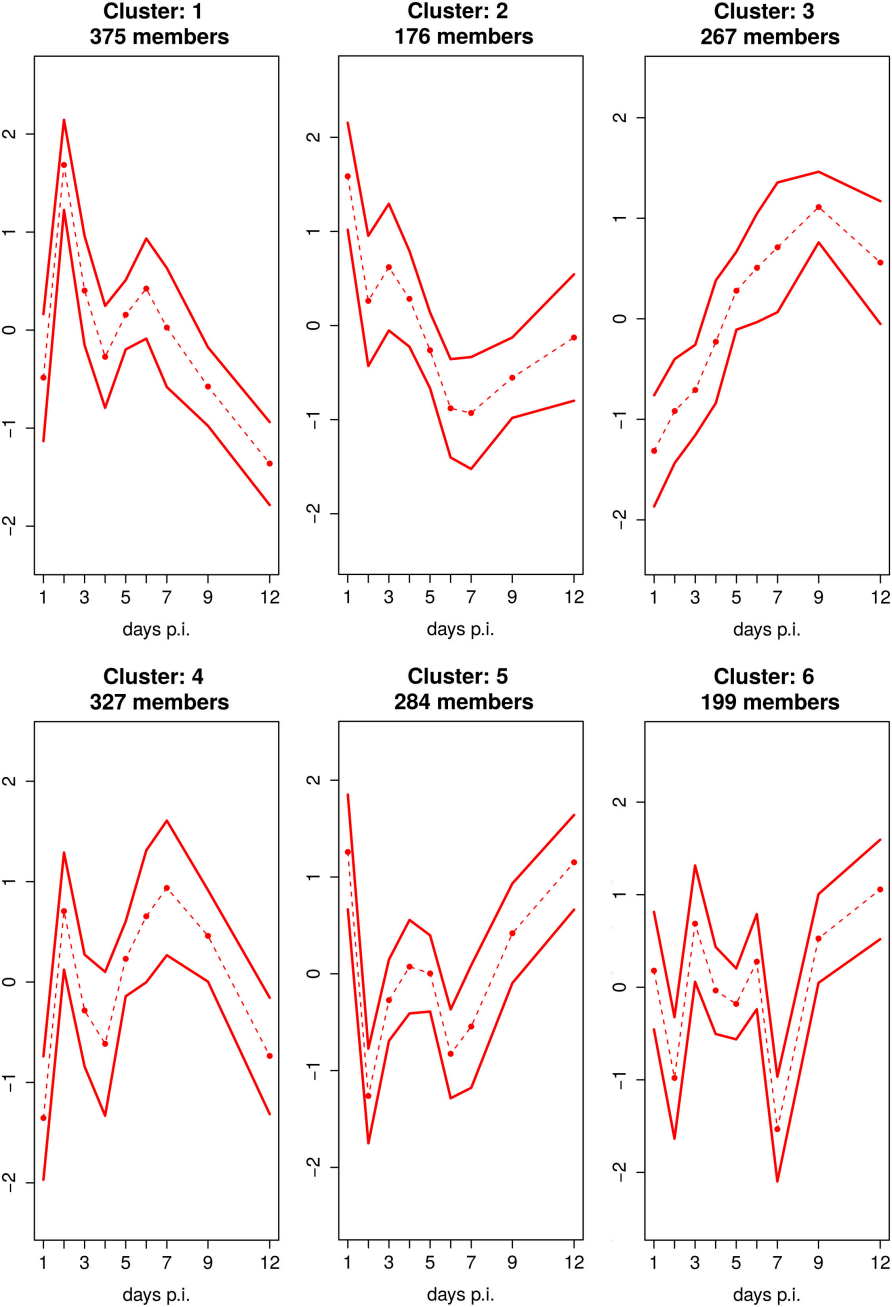
**Fuzzy c-Means Clustering of DEGs Reveals 6 Cluster**. Mean expression profile with standard deviation of 6 clusters, identified by fuzzy c-means clustering. The x-axis represents the day's p.i. whereas the y-axis represents the mean scaled log_2_ fold change expression for the cluster.

Cluster analysis shows that 6 clusters optimally represent the dynamics in the dataset (Figure 4, Supplementary Figure 2). The detailed GO enrichment analysis of the entire 6 clusters is presented in the Supplementary Table 4.

Five of the 6 clusters, i.e., the clusters 1, 2, 4, 5, and 6 comprising the majority of 1185 DEGs (representing 83% of all DEGs), are characterized by a distinct biphasic temporal expression pattern over the time course of infection showing strong up- (cluster 1 and 4) or down-regulation (cluster 2, 5, and 6) at day 2 p.i. which is followed by a stagnant recovery phase and further up- and/or down-regulation at day 6 or 7 p.i. In addition, Cluster 2, 5, and 6 show upregulation peaks e.g., at the day before and/or after the disease peaks.

Clusters 1 and 4, showing strong biphasic up-regulation, are being associated with the significantly overrepresented GO category ‘Immune response’ (*p* = 1.3 × 10^−22^ and 6.6 × 10^−23^, respectively; Supplementary Table 4). Cluster 1 contains 375 genes that were significantly up-regulated in the early phase of the host response with a strong first peak at day 2 p.i. and a second peak of lower intensity at day 6 p.i. followed by an increase of mostly down-regulated genes from day 7 p.i. on. Interesting genes involved in this cluster are e.g., Ifnb1, Il1a, Il1b, Myd88, Rsad2, Oas1a, Mx1, which are associated with the detection of virus and activation of innate immune responses by antiviral type I IFNs. In accordance, the GO enrichment analysis of genes belonging to cluster 1 showed that this cluster mainly comprised the GO terms “inflammatory response” and “response to virus.” Cluster 4 was associated with 327 genes that were strongly activated on day 2 as well as on days 6 and 7 p.i. Genes within this cluster are e.g., Ccl-3/-4/-5, Cxcl-9/-13/-16, Stat-1/-4, Socs1, Nfkbid. They are assigned to the overrepresented GO terms “Immune response,” “positive regulation of immune response,” “inflammatory response,” “response to wounding,” or “regulation of cytokine production.” Both cluster 1 and cluster 4 are enriched with many cytokines and chemokines genes (Supplementary Figure 3A) with biphasic behavior. Cluster 2 consists of 176 genes showing a strong upregulation at day 1 p.i., weak down-regulation at day 2 p.i. and a second peak of even stronger downregulation at day 6 p.i. and 7 p.i. GO enrichment analysis reveals that genes belonging to this cluster are associated with haemopoietic or lymphoid organ and blood vessel development. Cluster 5 and cluster 6 consists of 284 and 199 genes, respectively. Both clusters are also characterized by two strong down-regulation peaks of DEGs at days 2 and 7 p.i., which allocation coincides with temporal location of the peaks of the clusters 1 and 4. For cluster 5, the early peak was somewhat stronger than the second one, whereas the vice-versa was found for cluster 6. In addition, upregulation was detected in cluster 5 at day 1 p.i. as well as in cluster 5 and 6 at day 12 p.i. These two clusters are enriched with the GO terms “cell projection morphogenesis,” and “cell morphogenesis,” “epithelium development,” respectively (Supplementary Table 4). Genes with biphasic expression profiles belonging to the Clusters 1 and 4 as well as 5 and 6 also contained a number of reactive oxygen species (ROS)-related genes. Higher expression of pro-oxidation genes such as Ncf4 and Xdh and down-regulation of anti-oxidation genes such as Dhdh and Cat have been found (Supplementary Figure 3B).

Cluster 3 consists of 267 genes that are more or less continuously up-regulated until day 9 p.i. e.g., E2f1, Clspn, Prc1, Dbf4, Kntc1. This cluster is enriched with the GO terms “cell cycle and division,” “positive regulation of T-cell,” “lymphocytes and leukocytes activation,” and “positive regulation of immune system process,” which also indicate an association of the activation of adaptive immune responses with disease pathology. Interestingly, the immunomodulating Ifng also belongs to this cluster. It is weakly upregulated on day 2 and about 4-times between days 5 and 8 p.i.

### Regulatory network of murine influenza infection

The gene regulatory network was constructed by integrating gene expression profile along with prior knowledge (see Section Materials and Methods), extracted from Pathway Studio 9.0 (Nikitin et al., 2003). Supplementary Table 5 shows the prior knowledge information, and their corresponding confidence score used for network inference. As the complexity of network structure increases exponentially with the number of genes involved, a small number of genes should be selected to be included in the network reconstruction.

The candidate genes selection was based on the significant overrepresented gene enrichment analysis (see Section Materials and Methods). A set of 20 DEGs, most of them belonging to the overrepresented GO term “response to the virus,” with *p*-value of 1 × 10^−5^, was considered as candidate genes (Supplementary Table 2). The corresponding function for each gene (Supplementary Table 6) was extracted from GeneCards (Chalifa-Caspi et al., 2004). The GO categories other than “response to virus” was extracted from DAVID (Huang da et al., 2009a,b). Four of the identified candidate genes have pairwise very similar functions and high correlation with each other: Oas1a/Oas1b, Mx1/Mx2, Ddx58/Ifih1, and H2Q7/H2Q8. Therefore, we searched among the huge number of genes highlighted by the DAVID and GeneCards for additional genes that are strongly associated with TLR and IFN-gamma signaling, as also the case for some of the aforementioned candidate genes. Thus, we included four genes (Stat-1/-3, Irf1, and Socs1), which were shown to play a crucial role in feedforward and feedback inhibition of interferon and TLR signaling during macrophage activation (Hu et al., 2008).

**Figure 5:**
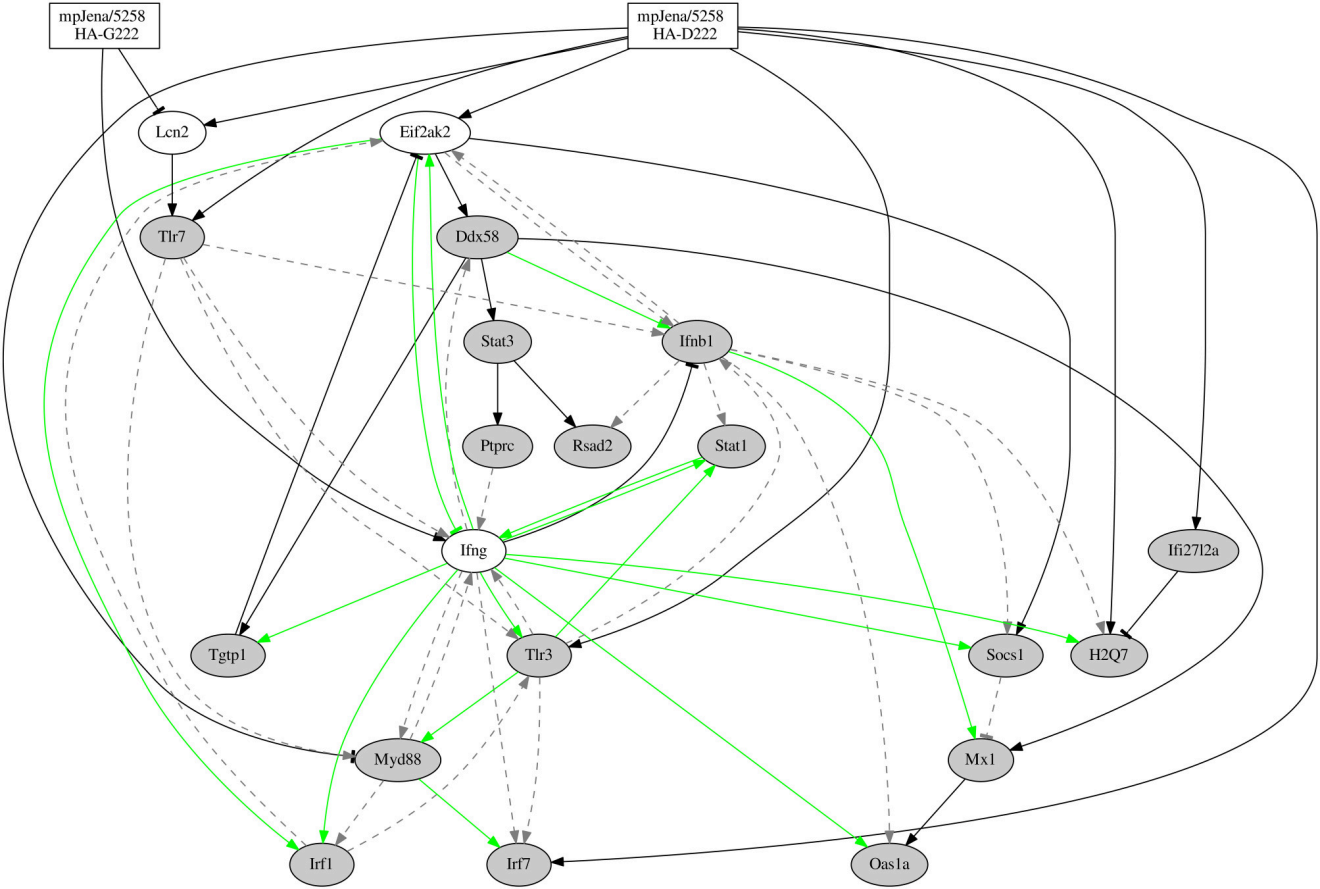
**Gene-regulatory network predicted from time series microarray data and prior knowledge**. Network for 20 genes (Supplementary Table 6), 16 of them selected through GO category “response to virus.” “Jena/5258 HA-D222” and “Jena/5258 HA-G222” represent the two influenza variants found by Seidel et al. (2014). Black edges represent the newly predicted edges by the NetGenerator tool, green edges represent edges supported by prior knowledge and confirmed by NetGenerator exploiting the expression data and gray dotted edges represent prior knowledge not confirmed by NetGenerator. Arrow-head represents activation or positive regulation while bar-head represents repression or negative regulation (that may also represent indirect interaction).

The final stable regulatory network is presented in Figure 5, which simulated kinetics fit to the expression profiles of the selected DEGs (Supplementary Figure 4). The network (Figure 5) considers the two mpJena/5258 variants (HA-D222 and HA-G222) that define the two input (perturbation) functions (Supplementary Figure 1) and 37 edges representing 27 gene-to-gene interactions and 10 influences of the influenza variants on the gene expression. A subset of 15 of the gene-to-gene interactions (colored green in Figure 5) were previously reported and were introduced as prior knowledge as shown in the Supplementary Table 5 extracted by Pathway Studio (Nikitin et al., 2003). Among the remaining 12 gene-to-gene interactions, 10 were novel interactions predicted by the inferred network. The other 2 were previously described in literature, but not used as prior knowledge for the network inference: Ddx58 positively activating Stat3 and Mx1 (Zeng et al., 2007; Breuer et al., 2013; Zhang et al., 2013). Among the 37 edges, we found 5 repressions or inhibitory effects and 32 activations among the overall network.

### Inflammatory subnetwork is robust

**Figure 6:**
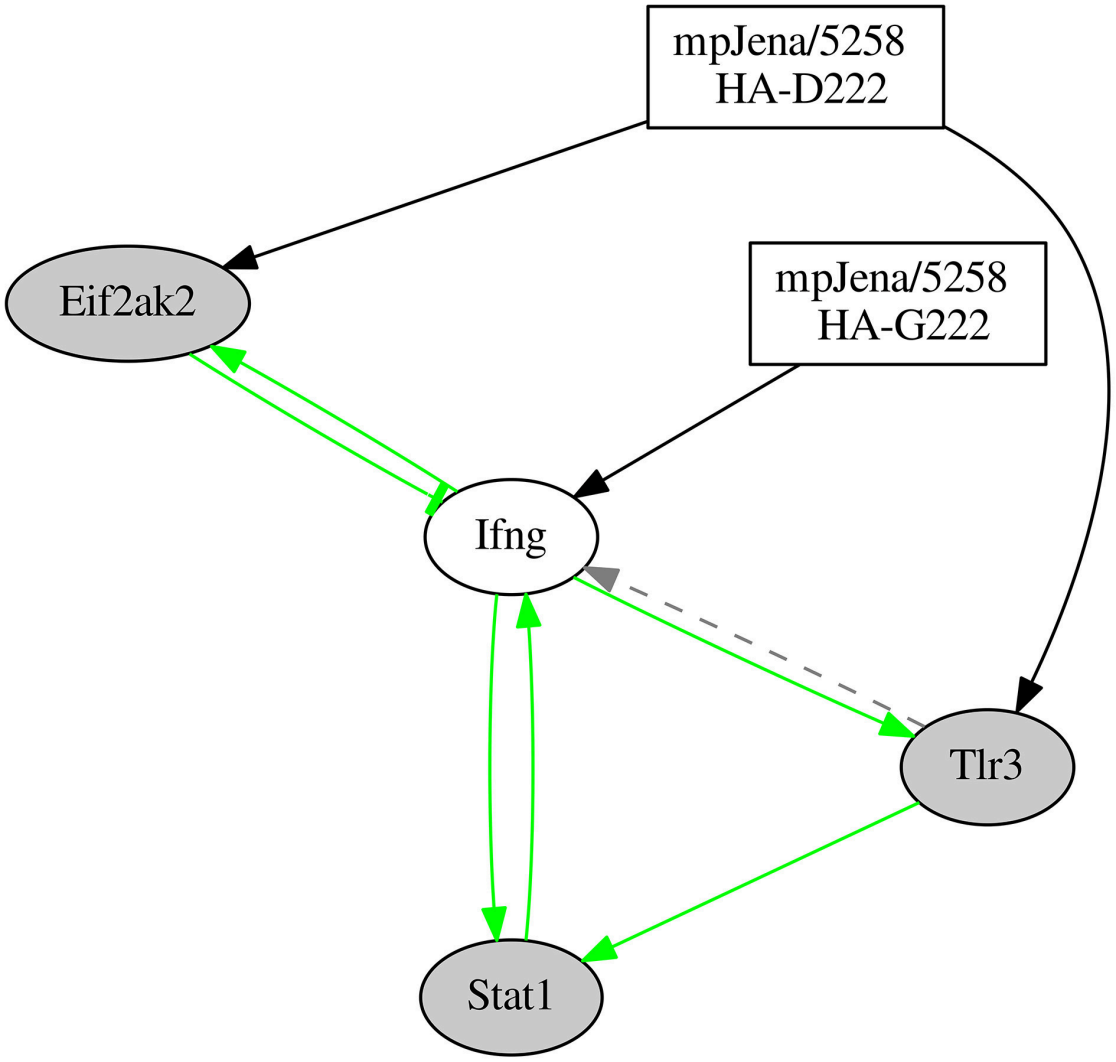
**Network predicted involving the 4 genes which were part of the positive feedback loop of the gene regulatory network**. “Jena/5258 HA-D222” and “Jena/5258 HA-G222” represent the two influenza variants found by Seidel et al. (2014). Black edges represent the newly predicted edges by the NetGenerator tool, green edges represent edges supported by prior knowledge and confirmed by NetGenerator exploiting the expression data and gray dotted edges represent prior knowledge not confirmed by NetGenerator. Arrow-head represents activation or positive regulation while bar-head represents repression or negative regulation (that may also represent indirect interaction).

As a part of the inferred network shown in Figure 5, a positive feedback loop formed by the interaction of Tlr3 − Ifng − Stat1 was discovered. To prove the stability of this loop, the robustness of this sub-network involving the positive feedback loop was investigated. We constructed two differential sub-networks involving four genes (Tlr3 − Ifng − Stat1 − Eif2ak2; Figure 6 and Supplementary Figure 5) and six genes (Tlr3 − Ifng − Stat1 − Eif 2ak2 − Socs1 − Ifi27l2a; Supplementary Figures 6, 7) adding one or three genes respectively, that are connected via robust edges with the 3-gene-loop. For the robustness analysis, we used the same algorithm as described above to construct the network from the gene expression data (see Materials and Methods). The robustness analysis for the 4-gene-subnetwork was performed by adding Gaussian noise of mean 0 and standard deviation (sd) of 0.01 and repeated network inference procedure for 500 times. The robustness analysis reveals that all the nine interactions were robust, i.e., the edges were recovered with a rate of 90% at least. The results of robustness analysis under the more stringent condition *sd* = 0.1 is shown in Supplementary Figure 8. Thus, all inferred edges shown in Figure 6 has been confirmed (with recovery rate >50%). The 4-gene-model simulation is shown in the Supplementary Figure 5. Similar analysis was done for the 6-gene-subnetwork with *sd* = 0.01. The robustness analysis also reveals remarkably high percentage (recovery rate ≥90%) for 13 out of 14 interactions involved, with an exception of an edge representing the influence of Influenza-2 variant to the gene Socs1 (62%). The 6-gene-subnetwork and simulation is shown in Supplementary Figures 6, 7. These results demonstrate that the positive feedback loop formed by the genes Tlr3 − Ifng − Stat1 is stable.

### High association between phenotypic and genotypic data

To find associations between the disease course and the corresponding whole genome expression data, we used multiple linear regression modeling. Here, the clinical (symptom) score S observed after infection of BALB/c mice with mpJena/5258 was used as dependent response variable as described recently (Manchanda et al., 2014). The scaled gene expression ratio values of the four genes of the 4-gene- subnetwork (i.e., Ifng, Stat1, Tlr3, Eif2ak2) were used as dependent (explanatory) variables. The linear regression model is shown in Materials and Methods. Interestingly, we found that the individual influence of the four individual independent variables is not significant (*p* ≥ 0.3 for S~ Ifng, S~ Stat1, S~ Tlr3, and S~ Eif2ak2) whereas a significant influence was found for the combined influence of the four genes on the clinical score (overall *p*-value 0.024 for S~ Ifng + Stat1 + Tlr3 + Eif2ak2 − 1). These results suggest that the phenotype of the disease is controlled by overall interactions between these independent variables rather their individual expression.

## Discussion

Interestingly the dynamics of about 83% of DEGs was characterized by biphasic up- and/or down-regulation after infection of mice with the A(H1N1)pdm09 mpJena/5258 consisting HA-222D/G quasispecies. This biphasic pattern was confirmed by the plasma levels of IFN-γ and IL-6, two key inflammatory cytokines that are associated with virus-induced inflammatory immune responses (Pommerenke et al., 2012; Goraya et al., 2015; Shoemaker et al., 2015). Moreover, a gene regulatory network for 20 selected DEGs was inferred to hypothetically explain the biphasic pattern caused by the coinfection with HA-222D/G quasispecies. The network results suggest that a positive feedback loop of Ifng, Stat1, and Tlr3 gene signaling is triggered by the HA-G222 variant.

As already published (Pommerenke et al., 2012) and based on our model study exploiting the clinical score comparing different virus strains by mathematical modeling (Manchanda et al., 2014), we primarily assumed to identify genes with expression profile maxima close to the early maximum of the clinical score and another set of genes whose temporal expression maxima explain the later peak of the clinical score. However, this working hypothesis failed. Hence, we did not identify two sets of genes, one with an early peak (caused by innate immunity) and the other one with a late peak (caused by adaptive immunity) in the measured expression profiles. In contrast, the majority of DEGs was characterized by biphasic behavior itself (up and/or down regulated DEGs as seen in cluster analysis). This biphasic behavior was also detected on the protein level for IFN-γ and IL-6. Based on these results the hypothesis predicted by modeling (Manchanda et al., 2014) needs modification.

A common feature of severe influenza is the strong immune response, characterized by activation of epithelial cells, macrophages and the recruitment and activation of neutrophils, eosinophils, monocytes, and further immune cells as reviewed in Iwasaki and Pillai (2014) and Goraya et al. (2015). Activated immune cells are known to generate highly reactive ROS (Rahman, 2003). ROS lead to lipid peroxidation and increase of tissue permeability which have been implicated in the pathogenesis of lung injury. Here, the genome expression profile confirms a strong expression of pro-oxidation genes such as Ncf4 and Xdh along with down-regulation of anti-oxidation genes such as Dhdh and Cat. This strong pro-oxidation stimulation may increase lung injury and the severity of the disease. In agreement with our findings, high levels of expression of Ifng, IL1, IL6, Ccl4, Ccl5, Cxcl9, and Cxcl10 in the lungs of infected mice which were associated with enhanced pathogenicity in previous studies (Kobasa et al., 2004; Tumpey et al., 2005). The proven strong cytokine and chemokine gene transcription (overrepresented in gene cluster 4) may result from but also trigger the immune cell infiltration that has been seen in the lungs of mpJena/5258-infected mice analyzed here (Seidel et al., 2014).

The crucial role of viral HA for enhanced pathogenicity was demonstrated with recombinant viruses expressing the HA of a 1918 pandemic H1N1 IAV (Kobasa et al., 2004). These recombinant viruses induced high levels of macrophage-derived chemokines and cytokines, which resulted in infiltration of inflammatory cells and severe hemorrhage (hallmarks of the illness produced during the H1N1 1918 pandemic) in mice. In the present study, we used an ordinary differential equation-based modeling approach to infer a gene regulatory network to hypothetically explain the correlation between the biphasic gene expression patterns with HA-222D/G quasispecies evolution and disease severity in mice. To our knowledge, this is the first gene regulatory network involving two variants (HA-D222 and HA-G222 quasispecies) of A(H1N1)pdm09. We inferred a network of 20 DEGs where 16 of them belong to the GO category “response to virus” and four of them were included based on the finding published elsewhere (Hu et al., 2008). Of course, this gene selection made for modeling is biased by prior knowledge and not comprehensive (i.e., genome-wide). Among the 12 predicted edges of the 20-gene network there are two gene-to-gene interactions, Ddx58 positively activating Stat3 and Mx1, which were previously described in literature (Zeng et al., 2007; Breuer et al., 2013; Zhang et al., 2013), but not used as prior knowledge for the network inference. This confirms the predictive power of modeling, despite the low quality of model fit for the respective genes.

The inferred gene regulatory network comprises a positive feedback loop (supported by prior knowledge) between Stat1, Ifng, and Tlr3. The inference of this loop was confirmed to be robust in a reduced 4-gene-network. Recently, a high complexity for inflammatory networks, which is accompanied by high entropy and low free energy, was shown, for a highly pathogenic H5N1 IAV (Jin et al., 2014). This is in accordance with our regulatory network prediction where we found a complex regulation of Ifng (high degree of Ifng). Another recent study (Brandes et al., 2013) also described an elevated activation of inflammatory signaling networks in lethal IAV infection and a positive feedback chemokine-derived loop regulating the pro-inflammatory response. This is in concordance with our finding of a positive feedback loop between Ifng, Stat1 and Tlr3, where Ifng represents the pro-inflammatory gene. IFN-γ was particularly strong induced in the severe phase of disease. Furthermore, TLR3-IAV interaction critically contributes to a detrimental host inflammatory response (Le Goffic et al., 2006). While this manuscript was in progress, further studies demonstrated that cytokine levels are regulated in a time-dependent and IAV strain-dependent manner (Shoemaker et al., 2015). According to Shoemaker et al. “the dynamics of inflammation-associated gene expression are regulated by an ultrasensitive-like mechanism in which low levels of virus induce minimal gene expression but expression is strongly induced once a threshold virus titer is exceeded. A systematic exploration of the pathways regulating the inflammatory-associated gene response suggests that the molecular origins of this ultrasensitive response mechanism lie within the branch of the Toll-like receptor pathway that regulates STAT1 phosphorylation” (Shoemaker et al., 2015). This is in in good agreement with our results where network analysis showed that the upregulation of Tlr3, Ifng, and Stat1 was associated with disease severity. The positive feedback loop between Tlr3 − Ifng − Stat1 presented here was induced by the HA-G222 variant. This model-predicted induction warrants further experiments in mice individually infected with the different HA-222 quasispecies. Overall, our study shows a role of this positive feedback loop in triggering the pro-inflammatory response in the lung of mice coinfected with A(H1N1)pdm09 HA-222 quasispecies.

Finally, we also focused on the comparison between the virus-induced symptoms (clinical score and body weight changes, Manchanda et al., 2014) and the gene expression profile. A high correlation of the virus-induced symptoms with gene expression patterns and cytokine levels in the blood of the infected animals was shown.

In conclusion, the coevolution of HA-222D/G quasispecies of mpJena/5258 elicits a complex response in infected mouse lung, characterized by a biphasic gene expression pattern. Increasing amounts of the HA-G222 variant correlated with a substantial upregulation of Ifng via the positive feedback loop Tlr3 − Ifng − Stat1 at day 7 p.i. We hypothesize that this finally contributed to the observed strong pro-inflammatory response. The expression profiles of the genes Tlr3, Ifng, Stat1, and Eif2ak2 in the lung as well as the Il-6 and IFN-γ level in serum were biphasic like the virus-induced symptoms (Manchanda et al., 2014; Seidel et al., 2014). The present study may thus serve as an indicator to suggest that the occurrence of quasispecies provoking biphasic courses in the host's immune responses might be an interesting new aspect for further exploring and comparing the host response to IAV infections and for developing control strategies.

## Author contributions

HM, NS, RG, and MS designed research; HM, NS, HS, MB, RC, and JL performed research; HS, AS, and MS contributed new reagents/analytic tools; HM, RG, NS, HS, MB, RC, JL, AS, and MS analyzed data; All authors wrote and approved the paper.

## Funding

This study was supported by the European Social Fund and the Thuringian Ministry of Economy, Labour, and Technology [2011FGR0137 to MS]; by the Federal Ministry for Education and Science [BMBF 03Z2JN22 to HS]. Funding for publication/open access charge: Leibniz Institute for Natural Product Research and Infection Biology - Hans Knoell Institute, Jena, Germany.

### Conflict of interest statement

The authors declare that the research was conducted in the absence of any commercial or financial relationships that could be construed as a potential conflict of interest.
