# Supplementary material for: Differential Biphasic Transcriptional Host Response Associated with Coevolution of Hemagglutinin Quasispecies of Influenza A Virus
Source: Front Microbiol. 2016 Aug 3;7:1167. doi: 10.3389/fmicb.2016.01167 (PMC4971777; doi:10.3389/fmicb.2016.01167)
Supplement: Supplementary file 7 [file DataSheet1.pdf]

Supplementary Material

Differential Biphasic Transcriptional Host Response Associated with Coevolution of Hemagglutinin Quasispecies of Influenza A Virus

Himanshu Manchanda, Nora Seidel, Markus F. Blaess, Ralf A. Claus, Joerg Linde, Hortense Slevogt, Andreas Sauerbrei, Reinhard Guthke* and Michaela Schmidtke*

*** Correspondence:** Corresponding Author: michaela.schmidtke@med.uni-jena.de and reinhard.guthke@leibniz-hki.de

1 Supplementary Figures and Tables

1.1 Supplementary Figures

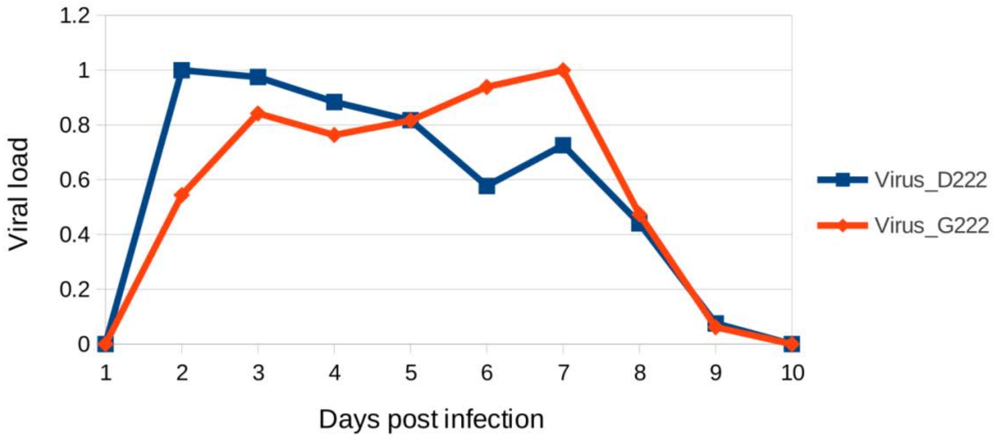

Supplementary Figure 1. Perturbation Function Profiles. The values are calculated by the viral titre values given by Seidel et al. (4), multiplied by the percentage value of HA-222D/G quasispeciesof mpJena/5258. The blue solid line represents mpJena/5258 HA-D222 variant and the orange solid line the mpJena/5258 HA-G222 variant.

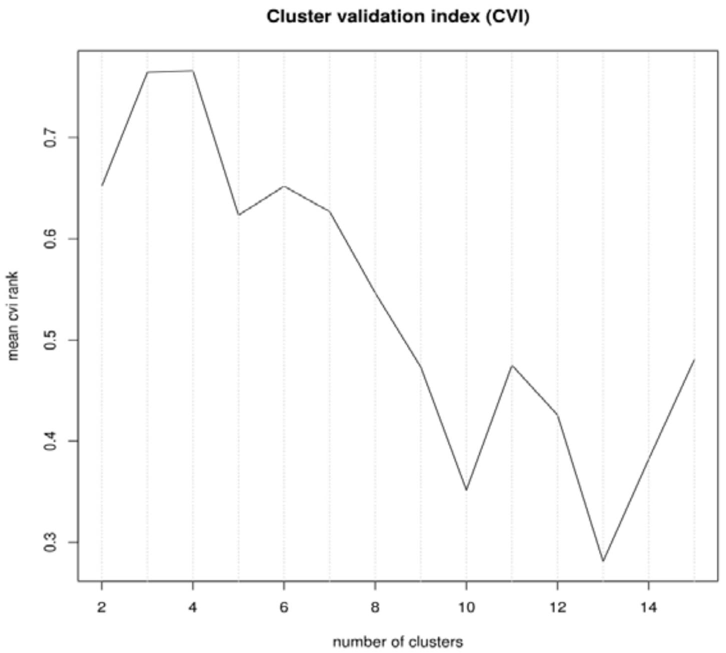

Supplementary Figure 2. Mean Cluster Validation Index (CVI). Values based on different cluster validation criteria including the Dunn index and Davis–Bouldin index. The x-axis represents the number of clusters and the y-axis represents the corresponding CVI values. Higher value of CVI represents good estimates of the cluster number. We found a number of 6 clusters quite stable with respect to the CVI value and they also cover most of the expression pattern of the DEGs over infection process.

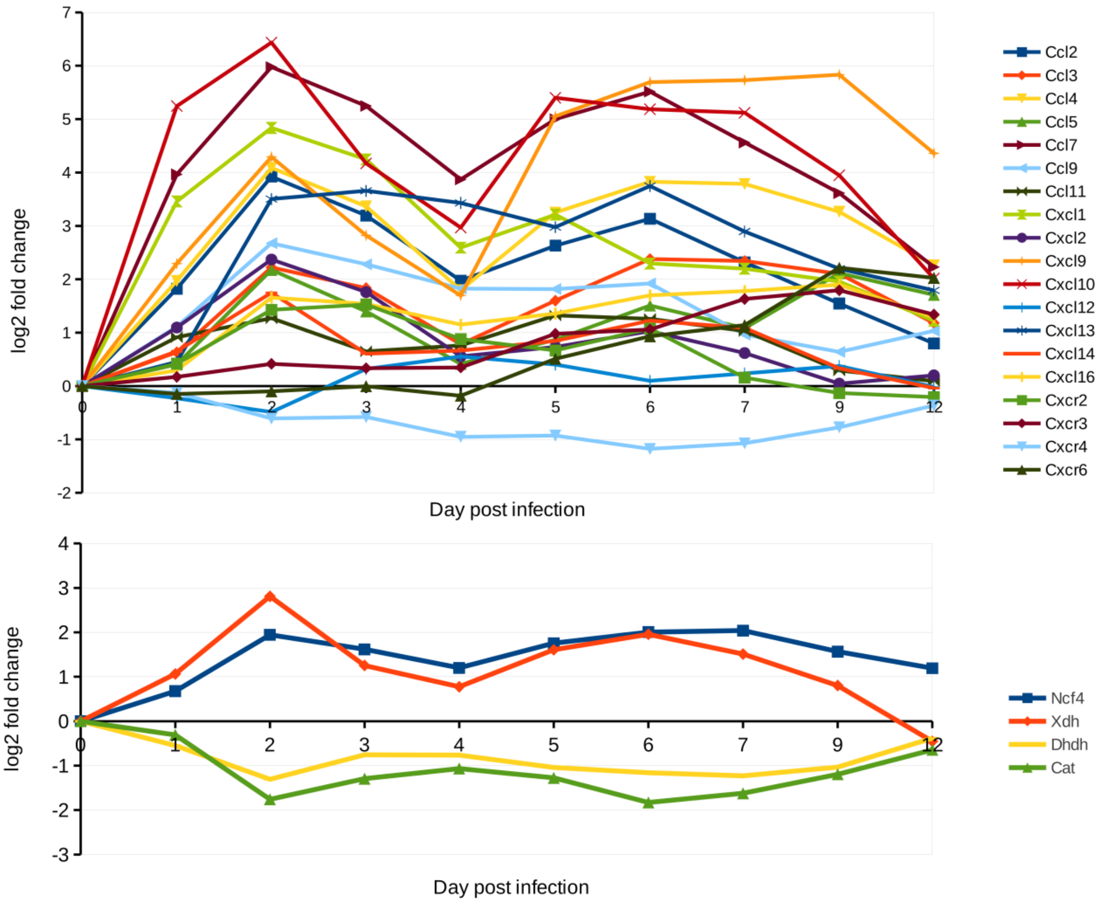

Supplementary Figure 3. Examples of Different Gene Expression Profiles. (A) Expression profiles of chemokines and their receptors. (B) Pro-oxidation genes (Ncf4 and Xdh) are upregulated and anti-oxidation genes (DhDh, Cat) are downregulated. The y-axis covers the expression changes, represented as compared to the mock-infected control mice on a log2 scale, whereas the x-axis represents the days p.i.

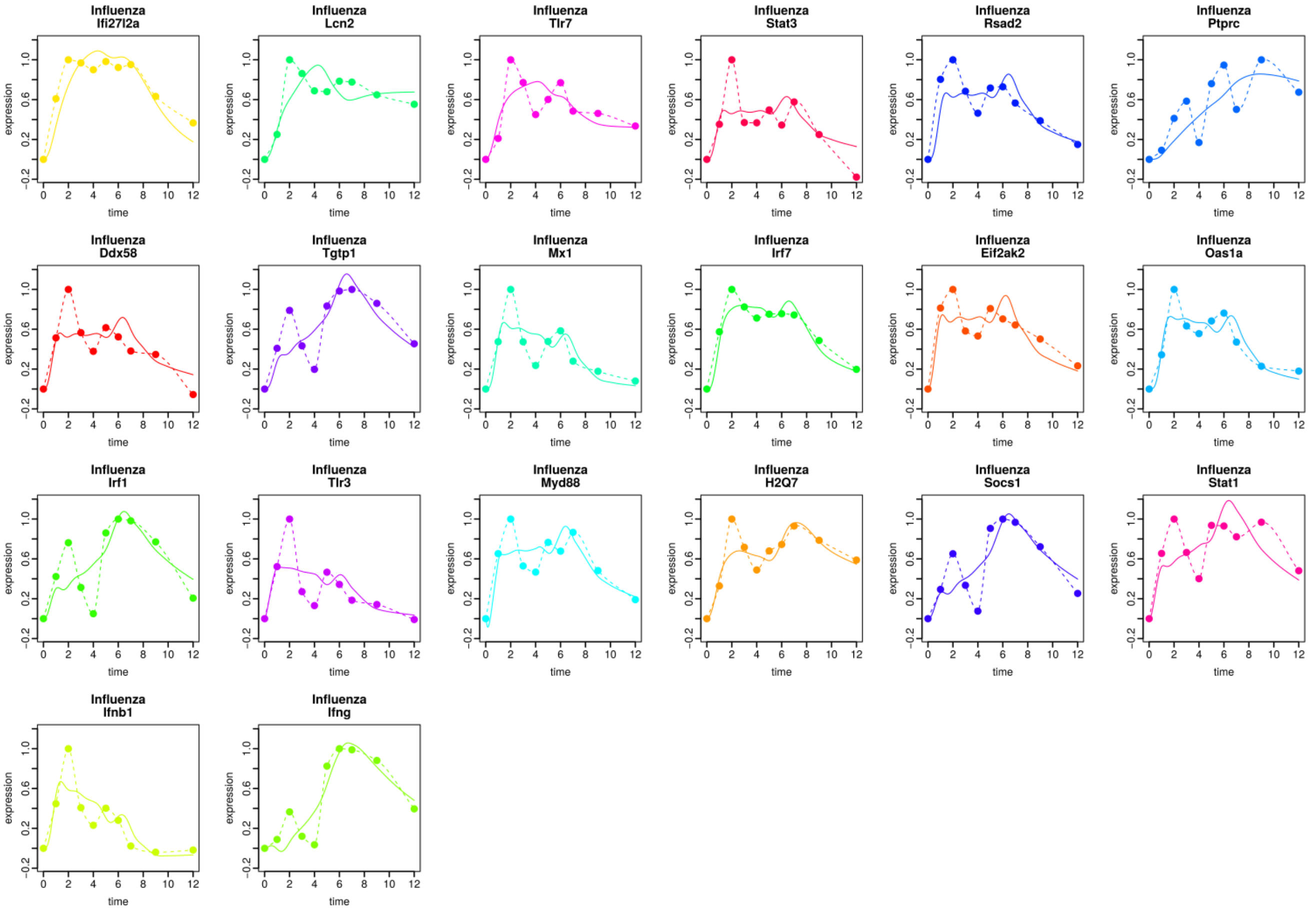

Supplementary Figure 4. Measured Gene Expression Profiles and Simulated Kinetics of the 20-Gene submodel. Output of the NetGenerator 2.0 R-package showing the simulated data fitted to the observed gene expression data of 20 selected genes. Dotted lines show the observed gene expression data over a period of 12 days, while the solid lines show the respective model fit. The x-axis shows the progression of infection over day's p.i., while the y-axis represents the gene expression changes (scaled $\log_2$ fold changes).

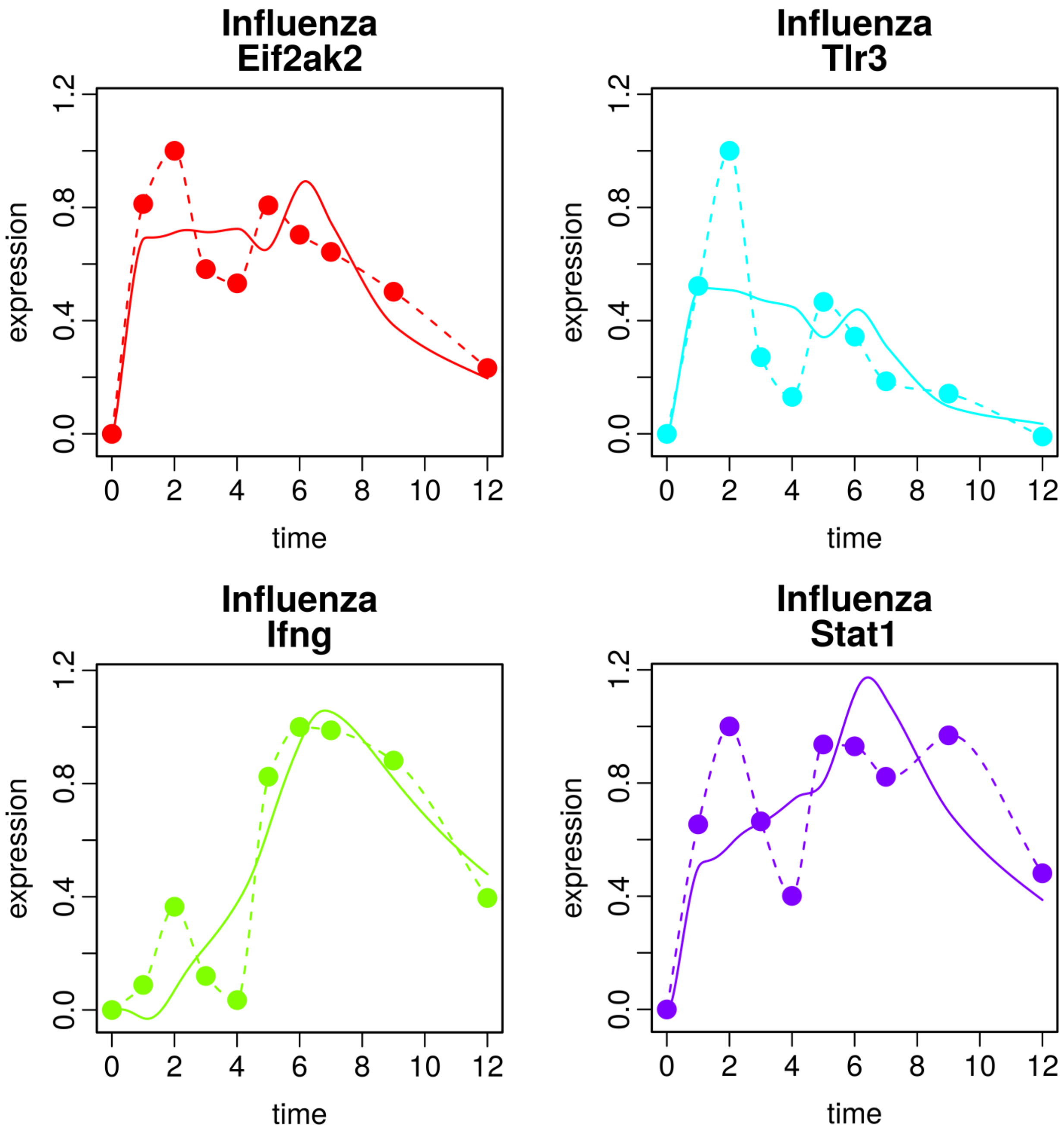

Supplementary Figure 5. Measured Gene Expression Profiles and Simulated Kinetics of the 4-Gene Submodel. Output of the NetGenerator 2.0 R-package showing how well the simulated data fitted to the observed gene expression data of 4 DEGs connected with the positive feedback loop as shown in result section. Dotted lines show the observed gene expression data over a period of 12 days, while the solid lines show the respective model fit. The x-axis shows the progression of infection over days p.i., while the y-axis represents the gene expression changes (scaled $\log_2$ fold changes).

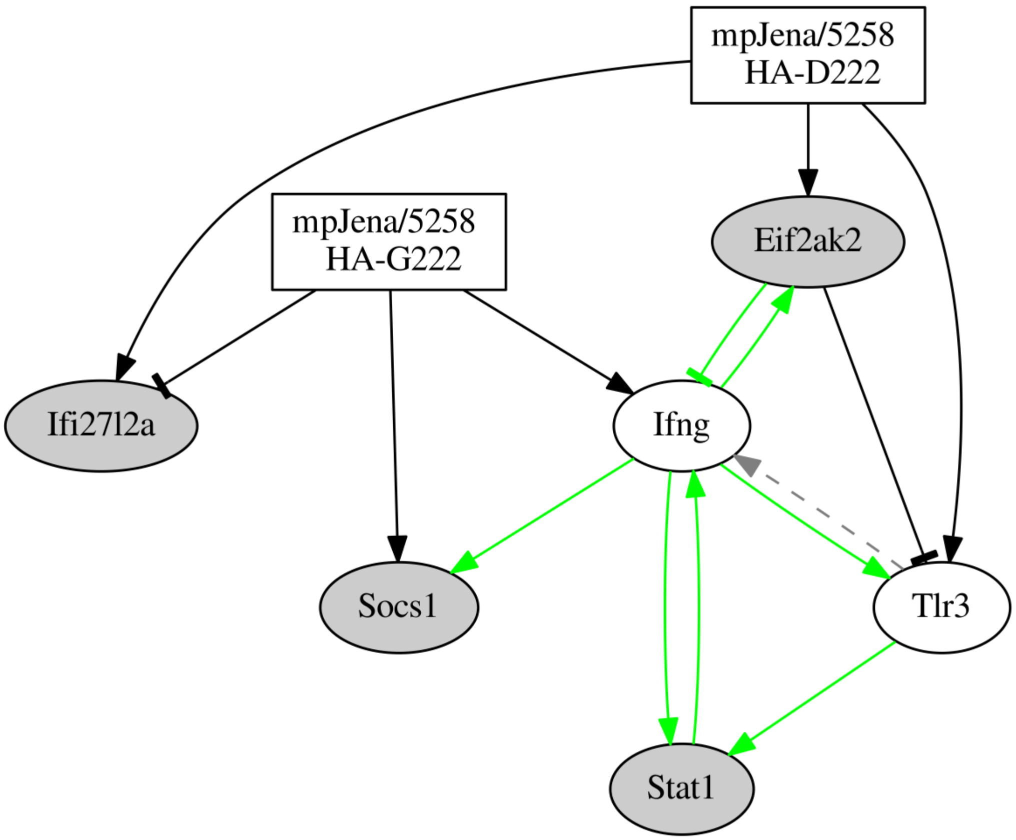

Supplementary Figure 6. Six-Gene submodel. Gene-regulatory network prediction from high throughput time series microarray data by NetGenerator 2.0 involving DEGs connected to the positive feedback loop. 'Influenza 1' and 'Influenza 2' represent the two influenza variants (mpJena/5258 HA-D222 and mpJena/5258 HA-G222, respectively). Black edges represent the newly predicted ones, green edges represent edges supported by the prior knowledge and confirmed by the expression data-based network inference and grey dotted edges represent prior knowledge not included in the network prediction based on the measured gene expression profiles. Arrow-head represents activation or positive regulation while bar-head represents repression or negative regulation (that may also represent indirect interaction).

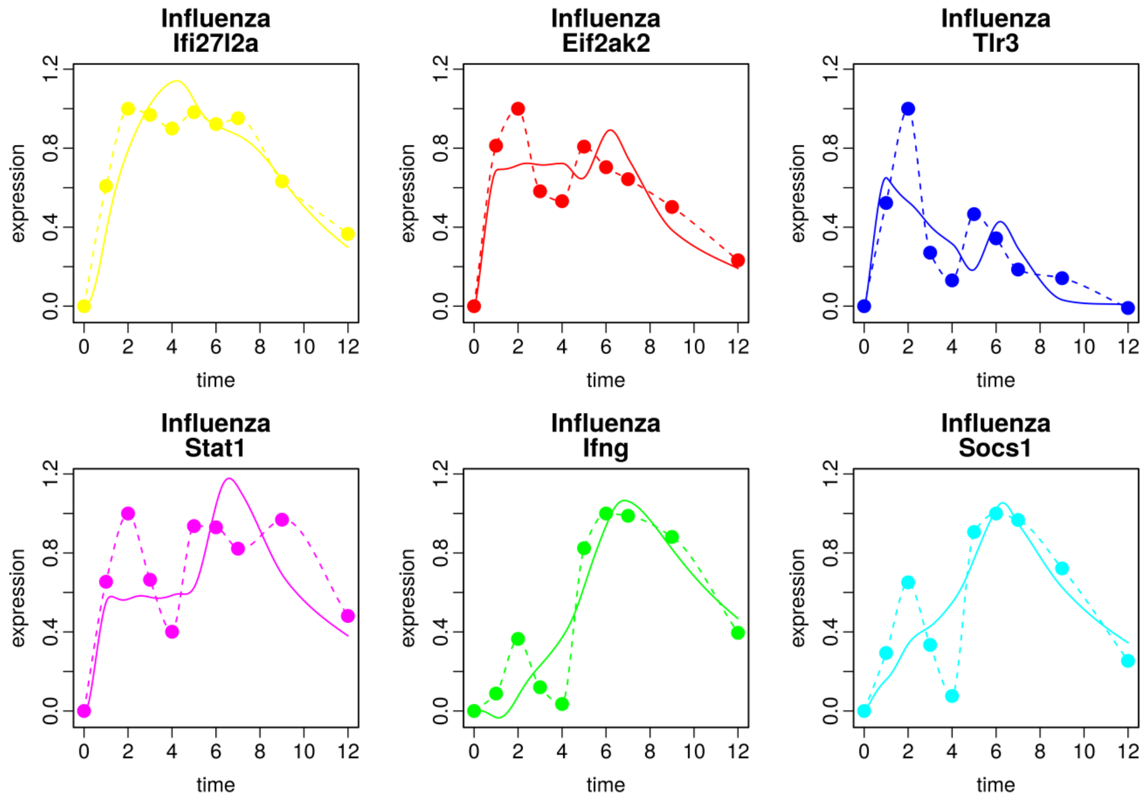

Supplementary Figure 7. Measured Gene Expression Profiles and Simulated Kinetics of the of the 6-Gene Submodel. Output of the NetGenerator 2.0 R-package, showing how well the data fitted to the observed gene expression data of 6 DEGs connected with the feedback loop as shown in result section. Dotted line shows the observed gene expression data for a gene over a period of 12 days, while the solid line shows the model fit. The x-axis shows the progression of infection over days p.i., while the y-axis represents the gene expression changes (scaled log2 fold changes).

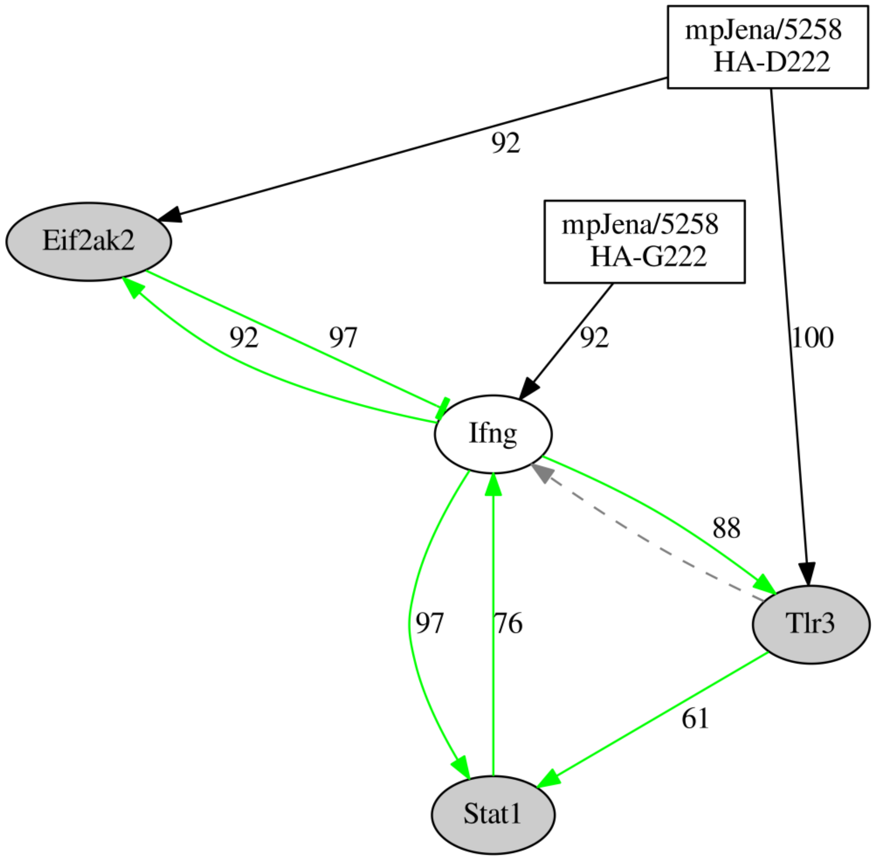

Supplementary Figure 8. Gene-regulatory Network Predicted from Time Series Microarray Data and prior Knowledge Involving the 4 Genes which were Part of the Positive Feedback Loop. In contrast to Fig. 5B with sd = 0.01 here more stringent condition sd = 0.1 is used for the robustness analysis.

1.2 Supplementary Tables

Supplementary Table 1. Differentially Expressed Genes (DEGs). Gene expression changes versus control (log2 fold change) in the lungs of BALB/c mice infected with influenza A virus mpJena/5258/09. A total of 1,628 DEGs were identified during the infection process of 12 days compared to controls.

- Uploaded as individual file due to large size of the EXCEL sheet

Supplementary Table 2. Overrepresented Functional Categories over all DEGs. A total of 1,628 DEGs identified during the infection process of 12 days p.i. compared to controls were mapped to significantly overrepresented functional categories using the DAVID tool.

- Uploaded as individual file due to large size of the EXCEL sheet

Supplementary Table 3. Interferon gamma (IFN-gamma) and Interleukin 6 (IL-6) Levels Detected in Serum. Sera were collected at different time points after infection of BALB/c mice with influenza A virus mpJena/5258/09. Each four serum samples per time point were analyzed for IFN-gamma as well as IL-6 by ELISA in two independent assays (I and II). Mean values were statistically analyzed.

d p.i.	Mouse no.	INF-gamma (pg/ml)			IL-6 (pg/ml)		
		I	II	mean[a]	I	II	mean[b]
1	M1	25.37	0	**12.69**	123.56	45.64	**84.60**
	M2	13.48	0	**6.74**	51.16	0	**25.58**
	M3	38.63	162.82	**100.73**	135.40	0	**67.70**
	M4	53.95	153.74	**103.85**	222.46	157.82	**190.14**
2	M7	183.08	291.52	**237.30**	1087.70	1121.40	**1104.55**
	M8	423.96	534.11	**479.04**	359.44	332.99	**346.22**
	M9	186.55	441.58	**314.07**	617.35	660.12	**638.74**
	M10	197.64	1012.90	**605.27**	364.09	366.21	**365.15**
3	M11	41.42	317.36	**179.39**	55.08	0	**27.54**
	M12	11.37	0	**5.69**	74.53	0	**37.26**
	M14	71.34	209.91	**140.63**	131.10	34.58	**82.84**
	M15	101.21	174.09	**137.65**	66.23	0	**33.11**
4	M16	2.22	0	**1.11**	64.01	0	**32.00**
	M17	13.48	0	**6.74**	0	0	**0.00**
	M18	5.75	171.99	**88.87**	170.12	96.43	**133.27**
	M19	8.56	175.77	**92.17**	21.99	0	**10.99**
5	M21	520.70	671.78	**596.24**	177.03	128.40	**152.72**
	M23	434.39	681.28	**557.84**	294.62	277.18	**285.90**
	M24	1028.00	1318.70	**1173.35**	1282.10	1308.20	**1295.15**
	M25	1313.20	1209.00	**1261.10**	425.43	456.48	**440.96**
6	M43	949.09	856.00	**902.55**	75.63	0	**37.82**
	M45	778.47	697.70	**738.09**	0	0	**0.00**
	M48	605.76	681.81	**643.79**	16.09	0	**8.04**
	M53	308.65	464.41	**386.53**	65.67	0	**32.84**
7	M32	251.75	853.68	**552.72**	3.21	0	**1.61**
	M35	187.93	296.11	**242.02**	0.55	0	**0.27**
	M39	299.62	450.96	**375.29**	0	0	**0.00**
	M40	189.32	318.74	**254.03**	12.50	0	**6.25**
9	M26	49.78	178.29	**114.03**	0	0	**0.00**
	M27	113.71	230.87	**172.29**	0	0	**0.00**
	M28	106.77	241.92	**174.35**	0	0	**0.00**
	M30	54.65	166.98	**110.82**	0.55	0	**0.27**
12	M31	2.22		**2.22**	3.86	0	**1.93**
	M34	9.97	0	**4.99**	0	0	**0.00**
	M41	22.58	0	**11.29**	0	0	**0.00**

	M44	30.96	153.33	**92.14**	0	0	**0.00**
Co	M62	0	0	**0.00**	0	0	**0.00**
	M64	0	0	**0.00**	0	0	**0.00**
	M67	2.20	0	**1.10**	0	0	**0.00**

[a]IFN-gamma statistics: p values determined by onefactorial ANOVA (Kruskal-Wallis)

**days p.i**	**1**	**2**	**3**	**4**	**5**	**6**	**7**	**9**	**12**
**2**	**0.045**								
**3**	0.566	0.154							
**4**	0.710	**0.018**	0.344						
**5**	**0.005**	0.402	**0.024**	**0.001**					
**6**	**0.007**	0.495	**0.035**	**0.002**	0.877				
**7**	0.065	0.877	0.204	**0.027**	0.321	0.402			
**9**	0.394	0.251	0.780	0.221	**0.047**	0.067			
**12**	0.609	**0.012**	0.278	0.889	**0.001**	**0.001**	**0.018**	0.172	
**Co**	0.222	**0.002**	0.080	0.381	**0.000**	**0.000**	**0.003**	**0.044**	0.455

[b]IL-6 statistics: p values determined by onefactorial ANOVA according to Kruskal-Wallis

**days p.i**	**1**	**2**	**3**	**4**	**5**	**6**	**7**	**9**	**12**
**2**	0.257								
**3**	0.753	0.148							
**4**	0.337	**0.036**	0.519						
**5**	0.329	0.875	0.197	0.053					
**6**	0.278	**0.027**	0.441	0.900	**0.039**				
**7**	0.078	**0.004**	0.148	0.422	**0.006**	0.499			
**9**	**0.018**	**0.000**	**0.041**	0.161	**0.001**	0.202	0.550		
**12**	**0.023**	**0.001**	**0.049**	0.186	**0.001**	0.232	0.604	0.937	
**Co**	**0.017**	**0.001**	**0.036**	0.134	**0.001**	0.166	0.449	0.838	0.782

Supplementary Table 4. Overrepresented Functional Categories over the DEGs Belonging to the 6 Clusters. In total, 6 different clusters of DEGs with similar expression profile were identified by cluster analysis. The genes from individual clusters were mapped to significantly overrepresented functional categories using DAVID tool.

- Uploaded as individual file due to large size of the EXCEL sheet

Supplementary Table 5. Prior Knowledge. Prior knowledge extracted from Pathway Studio 9.0 (3) for then 20 DEGs (Table 1) used for regulatory network inference.

- Uploaded as individual file due to large size of the EXCEL sheet

Supplementary Table 6. The 20 DEGs included in the network inference.

Genes	Function	GO Category other than "response to virus"	Cluster Association
**Ddx58** : Dead box protein (Asp-Glu-Ala-Asp)	1) Plays a major role in sensing viral infection and activation of antiviral responses including type-1 IFN and pro-inflammatory cytokines.<br><br>2) It is involved in viral double-stranded (ds) RNA recognition and the regulation of immune response.	Innate immune response.<br>Positive regulation of Ifn-alpha/-beta production.<br>Response to exogenous dsRNA.<br>RIG-I signalling pathway.	1
**Eif2ak2**: Eukaryotic Translation Initiation Factor 2-Alpha Kinase	1) serine/threonine protein kinase that is activated by autophosphorylation after binding to ds-RNA.<br><br>2) Also involved in the regulation of signal transduction, apoptosis, cell proliferation and differentiation.<br><br>3) Regulates various signalling pathways like NF-Kappa-b, insulin-signalling pathway and importantly, can regulates various Tfs like JUN, Stat1, Stat3, Irf1, Atf3.	-ve regulation of viral genome replication.<br><br>Positive regulation of chemokine n cytokine regulation.<br><br>Positive regulation of NF-kappa-b signalling.	1
**H2-Q7** or HLA-A: MHC Class I Antigen A	1) Involved in the presentation of foreign antigens to the immune system via MHC class I.	Antigen processing and presentation**.**<br><br>Positive regulation of T cell mediated cytotoxicity.	4
**Ifi27l2a**: Ifi27L2 IFN, alpha inducible protein 27-like 2	1) Important paralog of this gene is IfI27, which mediates IFN-induced apoptosis.	Aging.	1
**Ifnb1**: IFN beta 1<br>Type 1 IFN	1) Mainly involved in the signalling of Toll-like receptor signalling pathway and Immune response IFN alpha/beta signalling pathway.	Adaptive immune response.<br><br>Cytokine activity.<br>Defense response to virus.<br><br>B-cell proliferation and differentiation.	1
**Ifng**: IFN gamma<br>Type 2 IFN	1) The protein encoded is a soluble cytokine with antiviral, immunoregulatory and anti-tumor properties and is a potent activator of	Cytokine activity.<br><br>CD8-Positive, alpha-beta T-cell differentiation.	3

**Genes**	**Function**	**GO Category other than “response to virus”**	**Cluster Association**
	macrophages.	Positive regulation of IL-6, IL-12 biosynthetic process and<br>T-cell proliferation.	
**Irf1**: IFN regulatory factor 1	1) Activator of IFN alpha and beta transcription and also transcription activators of IFN induced genes like stat1.<br><br>2) Plays an important role in immune response directly affecting NK maturation and activity, macrophage production of IL12, Th1 development and maturation of CD8+ T-cells	Positive regulation of type 1 IFN & IL-12.<br>IFN-g mediated signalling regulation of Myd88-dependent TLR signalling.<br><br>CD8-Positive, alpha-beta T-cell differentiation.	4
**Irf-7**	1) play a role in the transcriptional activation of virus-inducible cellular genes, including Ifnb.<br><br>2) Can efficiently activate both the IFN-beta and the IFN-alpha genes and mediate their induction via both the virus-activated, MyD88-independent pathway and the TLR-activated, MyD88-dependent pathway.	Positive regulation of IFN-alpha/- beta production.<br><br>Regulation of Myd88 dependent TLR signalling pathway.	1
**Lcn2**: Lipocalin 2	1) Iron trafficking proteins involved in multiple processes such as apoptosis (due to IL3 deprivation), innate immunity and renal development.	Extrinsic apoptotic signalling pathway.<br><br>Iron ion binding.	4
**Mx1**: Myxovirus (Influenza virus) resistance 1 Disease associated: Influenza virus	1) GTP metabolising proteins that participate in cellular antiviral response.<br><br>2) Inhibits IAV replication by decreasing or delaying NP synthesis and by blocking endocytic traffic of incoming virus particles.	Innate immune response.<br><br>Response to virus.	1
**Myd88**: Myeloid differentiation primary response 88	1) Adapter protein involved in the Toll-like receptor and IL-1 receptor signalling pathway in the innate immune response.<br><br>2) Acts via IRAK1, IRAK2, IRF7 and	Positive regulation of IL-6, IL-17, IL-23.<br><br>Positive regulation of NF-kappa-B, JNK signalling.	1

Genes	Function	GO Category other than "response to virus"	Cluster Association
	TRAF6, leading to NF-kappa-B activation, cytokine secretion and the inflammatory response.	Regulation of inflammatory response.	
**Oas1a** n: Oas1: 2'-5' Oligoadenylate Synthetase 1 P52 Isoform	1) Interferon-induced, dsRNA - activated antiviral enzyme which plays a critical role in cellular innate antiviral response.<br><br>2) Mutations in this gene have been associated with host susceptibility to viral infection.	Negative regulation of viral process.<br><br>2'-5'- oligoadenylate synthetase activity.	1
**Ptprc** : Protein Tyrosine Phosphatase Receptor	1) PTPs are known to be signaling molecules that regulate a variety of cellular processes including cell growth, differentiation, mitosis, and oncogenic transformation.<br>2) Acts as a positive regulator of T-cell coactivation upon binding to DPP4	Activation of MAPK activity.<br><br>Negative regulation of viral genome replication.<br><br>Negative regulation of protein kinase activity.	3
**Rsad2**: Radical S-Adenosyl Methionine Domain-Containing Protein 2	1) a major role in the cell antiviral state induced by type I and type II interferon.<br><br>2) Displays antiviral activity against influenza A virus by inhibiting the budding of the virus from the plasma membrane by disturbing the lipid rafts.	CD4+, alpha-beta T-cell activation and proliferation.<br><br>Positive regulation of TLR-7 and TLR-9 signalling pathway.	1
**Socs1**: Suppressor of Cytokine Signaling 1	1) SOCS family proteins form part of a classical negative feedback system that regulates cytokine signal transduction.<br><br>2) SOCS1 is involved in negative regulation of cytokines that signal through the JAK/STAT3 pathway.	Kinase inhibitor activity.<br><br>JAK-STAT cascade.<br><br>Negative regulation of insulin-receptor signalling pathway	4
**Stat1**: Signal Transducer And Activator of Transcription 1	1) Involved in positive feed forward loop of IFN-gamma activation.<br><br>2) Acts as transcription activators and mediates the expression of a variety of genes important for cell viability in response to different cell stimuli and pathogens.	Cytokine mediated signalling pathway.<br><br>IFN-gamma mediated signalling pathway<br><br>Type-1 IFN signalling pathway.	4

**Genes**	**Function**	**GO Category other than “response to virus”**	**Cluster Association**
		Regulation of NF-KAPPA-B, JAK-STAT cascade	
**Stat3**: Signal Transducer And Activator of Transcription 3	1) Involved in negative feedback loop for IFN-gamma in combination with Socs1<br><br>2) plays a key role in many cellular processes like cell growth and apoptosis.	Involved in JAK-STAT cascade.<br><br>Negative regulation of cell death &<br>cell proliferation	1
**Tlr3**: Toll like receptor- 3	1) They recognize pathogen associated molecular patterns (PAMPs) and mediates the production of various cytokines.<br><br>2) It recognize dsRNA associated with viral infection and induces the activation of NF-kappa-B and production of type I interferons.	Positive regulation of NF-KAPPA-B, IFN-beta, IL-6, IL-12, JNK- cascade.<br><br>Positive regulation of type-I and type-III IFNs.<br><br>Positive regulation of NF-kappa-B signalling.	1
**Tlr7**: Toll like receptor- 7	1) Plays a role in pathogen recognition and activation of innate immunity.<br><br>2) Predominantly expressed in lung, placenta and spleen.	Positive regulation of IL-6, IFN-alpha<br><br>Defense response to virus.<br><br>Positive regulation of chemokine & IL-8 production and IFN-alpha/-beta synthesis	1
**Tgtp1**: T cell specific GTPase 1	1) It performs GTPase activity.	Response to IFN- alpha/-gamma.<br>cellular response to IFN-beta.<br>GTP catabolic process.	4
